# The Active Ingredients Identification and Antidiarrheal Mechanism Analysis of *Plantago asiatica* L. Superfine Powder

**DOI:** 10.3389/fphar.2020.612478

**Published:** 2021-01-19

**Authors:** Chun-Liu Dong, Yue Qin, Jin-Xin Ma, Wen-Qiang Cui, Xing-Ru Chen, Li-Ya Hou, Xue-Ying Chen, Bello-Onaghise God’spower, Nsabimana Eliphaz, Jun-Jie Qin, Wen-Xin Guo, Wen-Ya Ding, Yan-Hua Li

**Affiliations:** ^1^ College of Veterinary Medicine, Northeast Agricultural University, Harbin, China; ^2^ Heilongjiang Key Laboratory for Animal Disease Control and Pharmaceutical Development, Harbin, China; ^3^ Veterinary Medicine Engineering Laboratory, Beijing Centre Technology Co., Ltd., Beijing, China; ^4^ Heilongjiang Provincial Agricultural Products and Veterinary Medicine Technical Appraisal Station, Harbin, China; ^5^ College of Pharmacy, Guangxi University of Chinese Medicine, Nanning, China

**Keywords:** *Plantago asiatica* L., anti-diarrheal activity, superfine powder, active ingredients, luteolin, scutellarein

## Abstract

*Plantago asiatica* L. is a natural medicinal plant that has been widely used for its various pharmacological effects such as antidiarrheal, anti-inflammatory, and wound healing. This study aims to explore the antidiarrheal active ingredients of *Plantago asiatica* L. that can be used as quality markers to evaluate *P. asiatica* L. superfine powder (PSP). Molecular docking experiment was performed to identify the effective components of *P. asiatica* L., which were further evaluated by an established mouse diarrhea model. Na^+^/K^+^-ATPase and creatine kinase (CK) activities and the Na^+^/K^+^ concentrations were determined. The gene expression of *ckb* and *Atp1b3* was detected. PSP was prepared and evaluated in terms of the tap density and the angle of repose. The structures of PSPs of different sizes were measured by infrared spectra. The active ingredient contents of PSPs were determined by HPLC. The results indicated that the main antidiarrheal components of *P. asiatica* L. were luteolin and scutellarein that could increase the concentration of Na^+^ and K^+^ by upregulating the activity and gene level of CK and Na^+^/K^+^-ATPase. In addition, luteolin and scutellarein could also decrease the volume and weight of small intestinal contents to exert antidiarrheal activity. Moreover, as the PSP size decreased from 6.66 to 3.55 μm, the powder tended to be amorphous and homogenized and of good fluidity, the content of active compounds gradually increased, and the main structure of the molecule remained steady. The optimum particle size of PSP with the highest content of active components was 3.55 μm, and the lowest effective dose for antidiarrhea was 2,000 mg/kg. Therefore, the antidiarrheal active ingredients of PSP were identified as luteolin and scutellarein that exert antidiarrheal activity by binding with Na^+^/K^+^-ATPase. PSP was successfully prepared and could be used as a new dosage form for the diarrhea treatment.

## Introduction

*Plantago asiatica* L. (*P. asiatica*) belongs to the family of the Plantaginaceae, an annual or biennial herb, which is rich in resources and low in price in China. *P. asiatica* is widely distributed with more than 200 species (Najafian et al., 2018), and the medicinal properties of *P. asiatica* were recorded in early history books and the 2015 edition of the Pharmacopoeia. Besides, *P. asiatica* has been used in folk medicine for various pharmacological effects such as antidiarrhea and anti-inflammatory (Samuelsen et al., 1999).

Diarrhea is one of the leading causes of people morbidity and mortality in developing countries, especially in child (Black et al., 2019). The physiological mechanisms leading to diarrheal diseases are composed of accelerated intestinal transport, increased fluid volume in the intestinal lumen, and reduced absorption of water and electrolyte (Gorkiewicz et al., 2013) due to the changes in some enzymes and proteins. Na^+^/K^+^-ATPase is present in the basolateral membrane of small intestinal cells, providing a driving force for the active transport of many electrolytes (Bezerra et al., 2018). Inhibition of this intestinal enzyme may be critical for the regulation and absorption of Na^+^ and K^+^ in the intestine and leads to the accumulation of intestinal fluid, thereby contributing to diarrhea. According to other reports, creatine kinase (CK) could provide adenosine triphosphate (ATP) to the proper ATPase function which regulates Na^+^ and K^+^ levels (Baldissera et al., 2018). In addition, AQP4 is an aquaporin involved in water metabolism in the intestine, which, under expression inhibition, could cause the disturbance of water absorption and secretion, leading to diarrhea. Furthermore, the opioid receptor is related to the regulation of intestinal motility, which, with the combination of drug, could alleviate diarrhea by inhibiting intestinal peristalsis and prolonging intestinal retention (Fujita et al., 2014). Therefore, the regulation of these proteins may be an effective way on treating diarrhea.

Currently, superfine grinding technology has been widely used in manufacturing Traditional Chinese Medicine, which not only could destroy plant cell walls and promote the dissolution of the internal components (Tan et al., 2015) but also has many other unique advantages in the extraction of active ingredients (Zhao et al., 2015), such as simple operation, high mechanization, and pollution free. In the developed countries, superfine grinding technology has been widely used in medicine, cosmetics, and other fields. As a new dosage form, superfine powder possesses good dispersibility and solubility (Zhao et al., 2010), and the refinement of particles could increase the surface area and porosity (Tan et al., 2015). It has been found that superfine grinding could decrease bulk density, tapped density, and fluidity while improve the solubility (Zhao et al., 2015). However, to date, the effect of *P. asiatica* superfine powder (PSP) on diarrhea treatment has not been reported, and the antidiarrheal active ingredients and mechanism of *P. asiatica* on diarrhea treatment remain unknown.

Our preliminary results showed that the water extract of *P. asiatica* had a significant antidiarrheal effect, and the components, including baicalin and psyllium, were determined. Therefore, the present study aims to explore the antidiarrheal active ingredients of *P. asiatica* that can be used as quality markers to evaluate PSP. Moreover, the antidiarrheal activity and physical and chemical properties of PSP were determined, which provides a theoretical basis for the development of drugs for the treatment of diarrhea.

## Materials and Methods

### Materials and Animals

*Plantago asiatica* L. (3777555) was purchased from Traditional Chinese Medicine Market (Harbin, China). Luteolin, scutellarein, and caffeic acid were purchased from Solarbio Company (Purity ≥ 99%). Male or female clean Kunming mice (18–22 g) and clean Wistar rat (180–200 g) were obtained from the Department of Animal Center, Harbin Medical University (Harbin, China).

### Ethics Statement

All procedures used in this experiment were approved by the Institutional Animal Care and Use Committee of Northeast Agricultural University (No. NEAUEC20). Welfare-related assessments and interventions were carried out prior to, during, or after the experiments (Kilkenny et al., 2010).

### Ultraperformance Liquid Chromatography Coupled With Time-of-Flight Mass Spectrometry

The main chemical constituents in the effective parts of *P. asiatica* were identified by ultraperformance liquid chromatography coupled with time-of-flight mass spectrometry. The separation was performed on Waters Acquity UPLC BEH C 18 column (100 mm × 2.1 mm, 1.7 μm), and the mobile phase was eluted with a gradient of water A) containing 0.1% formic acid and acetonitrile B) containing 0.1% formic acid; Q-TOF/MS and electrospray ionization (ESI) sources were used for analysis in positive and negative ion modes; target search and nontarget search were performed by Peakview 2.0/masterview1.0 or Markerview 1.2.1 software. The formulation of the compound was then determined by the accurate mass and isotopic abundance ratio of the software target screening function. Their structures were determined by analyzing MS/MS fragments or comparing them to standard substances and references.

### Molecular Docking

#### Homology Modeling

The amino acid sequence of opioid receptors and AQP4 was retrieved from the UNIPRO database (http://www.uniprot.org). The BLAST protein database (http:/blast.ncbi.nlm.nih.gov/) performs a sequence similarity search on the PDB protein database. The model was generated using EasyModeller 4.0 (Pourseif et al., 2018).

##### Model Optimization and Evaluation

The protein structure is submitted to the Chrion (http://redshift.med.unc.edu/chiron/index.php) server for model optimization, and the optimized model was submitted to the structural analysis and verification server SAVES (http://servicesn.mbi.ucla.edu/SAVES/), which validate the model using the optional PROCHECK program (Bhattacharya et al., 2019).

##### CDOCKER Molecular Docking

The CHARMM-based CDOCKER method was used for molecular docking (Cui et al., 2019).

### Castor Oil-Induced Diarrhea

After being fasted for 18 h with free access to water, Kunming mice were randomly divided into seven groups (six per group). The first four groups were orally administered with scutellarein (1.75 mg/kg), caffeic acid (332.5 mg/kg), luteolin (5 mg/kg), and their mixture (including the three components mentioned above), respectively. The model group received 0.2 ml water, the positive group was given loperamide hydrochloride (2 mg/kg), and the negative group received ethanol (10%). Thirty minutes after the respective administration, 0.2 ml castor oil (voucher specimen number 20160920) was administered to each mouse to induce diarrhea. The mice without drug administer were used as the control group. The first diarrheal time, the total number of bowel movements, the number of loose stools, and the loose stools inhibition rate were calculated in 6 h. The inhibition rate of loose stools (%) = (A − B)/A × 100%, where A represents the mean loose stools number of model group and B represents the mean loose stools number of the drug group.

### Histopathology

The histological score was conducted following other report (Lu et al., 2018).

### Determination of Na^+^/K^+^-ATPase Activity, CK Activity, and Na^+^/K^+^ Concentrations in Small Intestine

Castor oil was used to induce diarrhea as described above. Groups 1 and 2 were administered scutellarein (1.75 mg/kg) and luteolin (5 mg/kg), respectively; groups 3 and 4 were administered the same volume of distilled water. One hour later, all groups received 1 ml castor oil except group 4. Three hours after the administration of castor oil, the animals were euthanized and laparatomized, and the small intestine (from the pylorus to the cecum) was separated and removed. The intestinal supernatant was prepared. The Na^+^/K^+^-ATPase activity and CK activity were determined according to Rat Na^+^/K^+^-ATPase (Na^+^/K^+^-ATPase) ELISA Kit (Bezerra et al., 2018) (Salarbio Co., Ltd.) and Rat Creatine Kinase ELISA Test Kit (Jiancheng Bioengineering Institution, Nanjing, China). The determination of Na^+^/K^+^ concentrations was carried out by the sodium kit and the potassium test box (Jiancheng Bioengineering Institution, Nanjing, China).

### Quantitative Real-Time PCR

The steps of total RNA extraction (Tiangen Biotech (Beijing) Co., Ltd) and cDNA synthesis (Takara) were performed according to the manufacturer's instructions. Primers used in quantitative real-time PCR are listed in Table 1 (Wang et al., 2020).

**TABLE 1:** Primers used for the quantitative RT-PCR analysis.

Gene	Primer sequence	
	Forward	Reverse
*ckb*	AGA​TGG​TGG​TGG​ACG​GAG​TGA​AG	TAG​GAA​GCG​GCA​GCC​TGG​TG
*Atp1b3*	GGG​TCT​CAT​CTT​GCT​CTT​CTA​C	CTT​CGG​AAC​CTC​GTC​ATT​CA
GAPDH	GAC​ATG​CCG​CCT​GGA​GAA​AC	AGC​CCA​GGA​TGC​CCT​TTA​GT

### Castor Oil-Induced Intestinal Fluid Accumulation

The castor oil-induced enteropooling assay was carried out. The Wistar rats were previously fasted for 18 h, with free access to distilled water, and randomly divided into four groups of six animals each group. Groups 1 and 2 received scutellarein and luteolin, respectively; groups 3 and 4 received distilled water, respectively. 1 h later, all groups received 1 ml castor oil except group 4. After 3 h, the animals were euthanized and laparatomized, and the small intestine was dissected from the pylorus to the cecum. The intestinal contents were collected into a graduated tube; then, the volume and weight were measured 5 times (Bezerra et al., 2018).

### Charcoal Meal Model of Intestinal Motility

Mice were fasted for 18 h with access to water arbitrarily and divided into three groups of six animals each group. Three groups received castor oil-induced diarrhea. After 1 h, each animal in group 1 was orally administered with scutellarein (1.75 mg/kg), each animal in group 2 was orally administered with luteolin (5 mg/kg), and each animal of group 3 received the same volume of distilled water. One hour after these treatments, the animals were orally given a charcoal suspension (0.2 ml/animal) containing 10% activated charcoal suspended in 5% Arabic gum. Thirty minutes later, the mice were euthanized, and the small intestine was immediately isolated. The travel distance of the charcoal meal and the total length of the intestine were measured 5 times, and the toner propulsion rate was expressed as the percentage of the distance traveled by the charcoal meal relative to the total length of the small intestine (Griggs et al., 2016).

### The Preparation of *P. asiatica* L. Superfine Powder and Laser Diffraction Analysis

*P. asiatica* was milled coarsely by ordinary shredder, and then, the resulting coarse samples were remilled by Taiwan’s original Taisaki FDV gas-type crushing Yusaki shredder. The powders were screened with five particle size distributions through changing the size, water content, and quality of samples before milling and then measured by a laser diffraction instrument (Niu et al., 2017). The appearance quality traits of samples were recorded.

### Scanning Electron Microscope

Morphological characterization of *P. asiatica* superfine powder was obtained using a scanning electron microscope (SEM), Quanta 200 FEG-SEM (FEI Co. Netherlands) on an image obtained at an accelerating voltage of 150 kV and a working distance of 10–15 mm. The samples were coated with platinum of 10 nm thicknesses to make the sample conductive (Zhao et al., 2010). Fifty particles were selected to determine the particle size and measured and calculated using the scale of the microscope software.

### Test Procedure for the Tap Density and the Angle of Repose

A 10 g sample was placed in a graduated cylinder and vibrated to a constant volume, and the final volume was recorded (Zhao et al., 2015). The tap density of PSP was calculated as follows:

$${\text{D}}_{0}\;\left(\text{g/ml}\right)=10\text{/V,}$$

where V was the final volume of PSP. All measurements were performed 5 times.

The angle of repose (θ) is defined as the maximum angle at which a pile of powder surface faces the plane supporting it. The angle of repose was measured using the sequence of steps stated here. First, a filler was fixed over some of the graph paper so that the distance (H) between the paper and the outlet of the filler outlet was 3 cm, and the filler was perpendicular to the paper. The different particle size powders were then separately poured into the filler until the tip of the powder cone contacted the outlet of the filler. The diameter (2R) of each type of powder was measured (Zhao et al., 2010). The angle of repose (θ) was calculated as follows:

θ= arctg (2H/R).

### Fourier Transform Infrared Spectroscopy

The sample was prepared by the potassium bromide (KBr) pellet method. A PerkinElmer Model GX Fourier Transform Infrared spectrophotometer (DTGS) was used at a temperature of 20°C. The spectra were recorded at a resolution of 4 cm^−1^ in the wavelength range of 4,000–400 cm^−1^, 32 scans, and a 2 cm^−1^ interval. The system was continuously purged with dry air. As a reference spectrum, the background spectrum of the same conditions was collected, but a medium (KBr) without PSP was collected (Zhao et al., 2015).

### Determination of Active Ingredient Contents of *P. asiatica* L. Superfine Powder

The active ingredient contents of PSP were determined, as described by Zhang et al. (2019a). The determination was carried out on a Thermo-C 18 column (4.6 mm × 250 mm, 5 μm) at a column temperature of 30°C. The mobile phase consisted of methanol A and 0.2% phosphoric acid water B. Flow rate was set at 0.6 ml/min. The gradient elution of scutellarein was 5–22% A at 0–3 min, 22–60% A at 3–15 min, 60–70% A at 15–20 min, 70–100% A at 20–30 min, and 100–5% A at 30–50 min. The gradient elution of luteolin was 55–62% A at 0–8 min, 62–70% A at 8–15 min, and 70–55% A at 15–35 min. The UV wavelengths were monitored at 335 and 350 nm, respectively. The injection volume was 10 μL.

### Acute Toxicity

There were some modifications to the acute toxicity test method compared to the previous method (Shang et al., 2018). The oral doses of PSP ranged from 900 to 10,000 mg/kg. The behavioral changes in the animals were continuously observed within 4 h, and then, the mortality was observed 24 h after the administration.

### Effect of *P. asiatica* L. Superfine Powder on Castor Oil-Induced Diarrhea

The effect of PSP on diarrhea induced by castor oil was investigated (Shang et al., 2018). Kunming mice were randomly divided into six groups (6 per group). Three doses of PSP (6,000, 4,000, and 2,000 mg/kg) were orally administered to each mouse; the model group received normal castor oil (10 ml/kg), the positive group was given loperamide hydrochloride (2 mg/kg), and the negative groups received distilled water. After 30 min, 0.2 ml castor oil (voucher specimen number 20160920) was administered to each mouse to induce diarrhea. The mice without drug administration were used as the control group. The evaluation indexes were the same as in the section of “Castor oil-induced diarrhea.”

### Data Analysis

All the experimental data were presented as mean ± SD. The statistical significance of the differences between the two groups was determined using SPSS Statistics 17.0 through Student’s t-test. Moreover, the pictures were plotted using GraphPad Prism 5.0 software. *p* < 0.05 was taken to indicate statistical significance.

## Results and Discussion

### Identification of *P. asiatica* Antidiarrheal Effective Components

**FIGURE 1:**
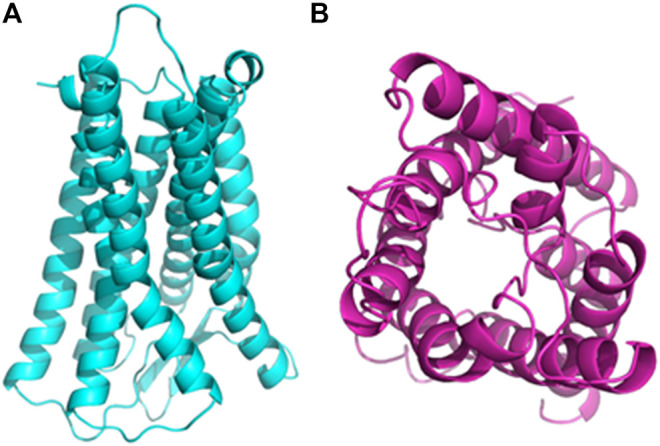
3D structural models. **(A)** The 3D structural model of opioid receptor. **(B)** The 3D structural model of AQP4.

**FIGURE 2:**
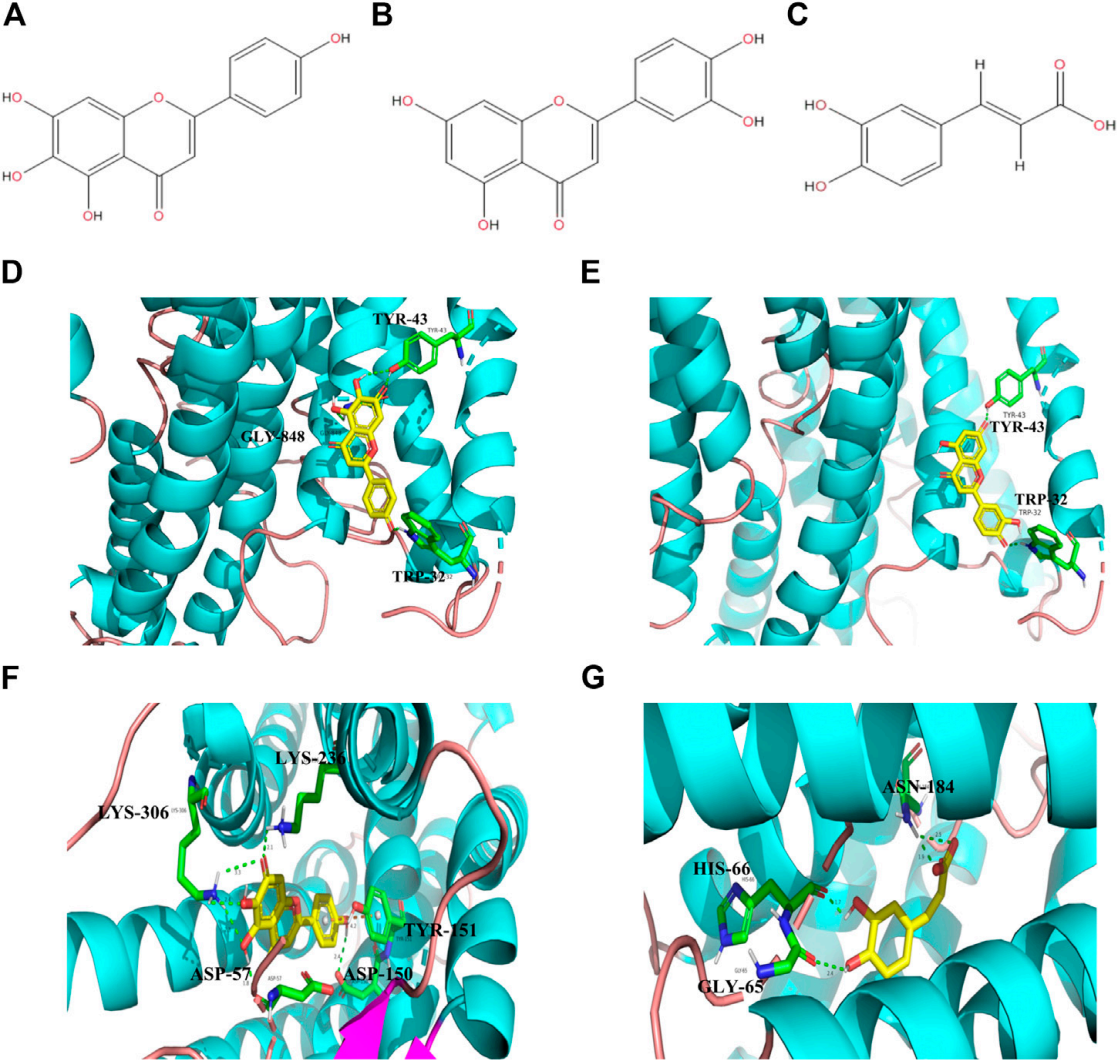
Computer simulation of molecular docking. **(A)** The structure of scutellarein. **(B)** The structure of luteolin. **(C)** The structure of caffeic acid. **(D)** Scutellarein combines with Na^+^/K^+^-ATPase. **(E)** Luteolin combines with Na^+^/K^+^-ATPase. **(F)** Scutellarein combines with opioid receptor. **(G)** Caffeic acid combines with AQP4.

In our previous research, the effective parts of *P. asiatica* were identified to be ethyl acetate and the residue parts (data not shown). Thereby, the UPLC-Q-TOF/MS test was carried out to identify the antidiarrheal effective components of the effective parts of *P. asiatica*. As a result, fourteen kinds of compositions, including plantamajoside, acteoside, homoplantaginin, 6-hydroxyluteolin-7-glucoside, baicalin, ursolic acid, martynoside, apigenin, scutellarein, caffeic acid, ferulic acid, luteolin, calceorioside B, and epimeredinoside A, were identified (Table 2). Computer-simulated molecular docking technology has been widely used in drug research and development (Zhou and Hua, 2020), which can simplify drug screening and reduce the complexity of drug screening. In order to identify the effective components of *P. asiatica*, molecular docking experiment was performed using three diarrhea-related proteins, Na^+^/K^+^-ATPase, opioid receptors, and AQP4 in this study (Table 3). The 3D structural model of opioid receptors and AQP4 was successfully established (Figures 1A,B). Moreover, out of fourteen components that are already known, three molecules with higher score were screened out, scutellarein, luteolin, and caffeic acid (Figures 2A–C). The results showed that scutellarein could form stable hydrogen bonds with amino acids TYP-43, GLY-848, and TRP-32 of Na^+^/K^+^-ATPase (Figure 2D), luteolin could form stable hydrogen bonds with amino acids TYR-43 and TRP-32 of Na^+^/K^+^-ATPase (Figure 2E), scutellarein could form stable hydrogen bonds with amino acids LYS-236, LYS-306, ASP-57, ASP-150, and TYR-151 of opioid receptors (Figure 2F), and caffeic acid could form stable hydrogen bonds with amino acids ASN-184, HIS-66, and GLY-65 of AQP4 (Figure 2G), indicating potential antidiarrhea activities.

**TABLE 2:** UPLC-Q-TOF/MS of two effective parts containing ethyl acetate and remaining parts of *P. asiatica*.

Components	RT (min)	Selected ion	Measured mass (m/z)	Calc. mass (m/z)	Primary secondary fragment ion (MS/MS) and source
Plantamajoside	5.5	[M + Na]^+^	663.186	663.18956	663[M + Na]^+^, 501[M + Na-C_6_H_10_O_5_]^+^, 483[M + Na-C_9_H_8_O_4_]^+^
Verbascoside	5.7	[M + Na]^+^	647.1923	647.19464	647[M + Na]^+^, 501[M + Na-C_6_H_10_O_4_]^+^
Pratensein-7-O-glucoside	6.2	[M + H]^+^	463.1207	463.12349	463[M + H]^+^, 301[M + H-C_6_H_10_O_5_]^+^, 286[M + H-C_6_H_10_O_5_-CH_3_]^+^
Hyperin	4.7	[M + H]^+^	465.10253	465.10275	465[M + H]^+^, 303[M + H-C_7_H_14_O_4_]^+^, 285[M + H-C_7_H_14_O_4_-H_2_O]^+^, 257[M + H-C_7_H_14_O_4_-H_2_O-CO]^+^, 169[M + H-C_7_H_14_O_4_-H_2_O-CO-C_7_H_4_]^+^
Baicalin	6.1	[M + H]^+^	447.0905	447.09219	447[M + H]^+^, 271[M + H-C_6_H_8_O_6_]^+^, 153[M + H-C_14_H_14_O_8_]^+^
Ursolic acid	18.4	[M + H]^+^	457.3643	457.36762	457[M + H]^+^, 411[M + H-CH_2_O_2_]^+^, 393[M + H-CH_2_O_2_-H_2_O]^+^, 203[M + H-CH_2_O_2_-H_2_O-C_14_H_22_]^+^
Martynoside	7	[M + Na]^+^	675.2223	675.22594	675[M + Na]^+^, 529[M + Na-C_6_H_10_O_4_]^+^
Apigenin	8.3	[M + H]^+^	271.0594	271.0601	271[M + H]^+^, 153[M + H-C_8_H_6_O]^+^, 145[M + H-C_6_H_6_O_3_]^+^, 119[M + H-C_7_H_4_O_4_]^+^
Scutellarein	5.3	[M + H]^+^	287.0542	287.05501	287[M + H]^+^, 269[M + H-H_2_O]^+^, 169[M + H-H_2_O-C_8_H_4_]^+^, 123[M + H-H_2_O-C_8_H_4_-CH_2_O_2_]^+^, 119[M + H-H_2_O-C_7_H_2_O_4_]^+^
Caffeic acid	5.4	[M + H]^+^	181.0483	181.04954	181[M + H]^+^, 163[M + H-H_2_O]^+^, 145[M + H-2H_2_O]^+^, 135[M + H-H_2_O-CO]^+^, 117[M + H-2H_2_O-CO]^+^, 107[M + H-H_2_O-2CO]^+^
Ferulic acid	7.4	[M + H]^+^	195.0642	195.06519	195[M + H]^+^, 177[M + H-H_2_O]^+^, 149[M + H-H_2_O-CO]^+^, 145[M + H-H_2_O-CH_4_O]^+^, 134[M + H-H_2_O-C_2_H_3_O]^+^, 117[M + H-2H_2_O-C_2_H_3_O]^+^
Luteolin	7.4	[M − H]^−^	285.0366	285.03936	285[M − H]^−^, 217[M-H-C_3_O_2_]^−^, 199[M-H-C_3_O_2_-H_2_O]^−^, 175[M-H-C_5_H_2_O_3_]^−^, 151[M-H-C_8_H_6_O_2_]^−^, 133[M-H-C_7_H_4_O_4_]^−^
Calceorioside B	1.3	[M − H]^−^	477.1567	477.13914	477[M − H]^−^, 315[M-H-C_6_H_10_O_5_]^−^, 161[M-H-C_6_H_10_O_5_-C_11_H_6_O]^−^, 133[M-H-C_6_H_10_O_5_-C_11_H_6_O-CO]^−^
Epimeredinoside A	7	[M − H]^−^	651.2877	651.22835	651[M − H]^−^, 505[M-H-C_6_H_10_O_4_]^−^, 475[M-H-C_6_H_10_O_4_-CH_2_O]^−^, 193[M-H-C_6_H_10_O_4_-CH_2_O-C_14_H_18_O_6_]^−^, 175[M-H-C_6_H_10_O_4_-CH_2_O-C_14_H_18_O_6_-H_2_O]^−^, 160[M-H-C_6_H_10_O_4_-CH_2_O-C_14_H_18_O_6_-H_2_O-CH_3_]^−^, 134[M-H-C_6_H_10_O_4_-2CH_2_O-C_14_H_18_O_6_-H_2_O-CH_3_]^−^

**TABLE 3:** Molecular docking scores for 14 components and opioid receptor, AQP4, and Na^+^/K^+^-ATPase.

Name	Score 1 (opioid receptor)	Score 2 (AQP4)	Score 3 (Na^+^/K^+^-ATPase)
Scutellarein	+22.384	+16.137	+22.957
Luteolin	+17.319	+18.063	+20.162
Caffeic acid	+16.107	+20.019	+17.847
Plantamajoside	+15.346	+18.814	+12.631
Verbascoside	+15.765	+14.223	+15.073
Pratensein-7-O-glucoside	+12.539	+13.826	+16.417
Hyperin	+18.765	+11.836	+15.071
Baicalin	+15.346	+17.154	+17.467
Ursolic acid	+14.643	+12.438	+14.682
Martynoside	+18.343	+14.463	+15.811
Apigenin	+18.424	+18.851	+14.668
Ferulic acid	+17.116	+15.474	+16.389
Calceorioside B	+13.111	+14.653	+15.146
Epimeredinoside A	+14.338	+16.856	+13.569

### Evaluation of the Antidiarrheal Activities of Luteolin, Scutellarein, and Caffeic Acid

**FIGURE 3:**
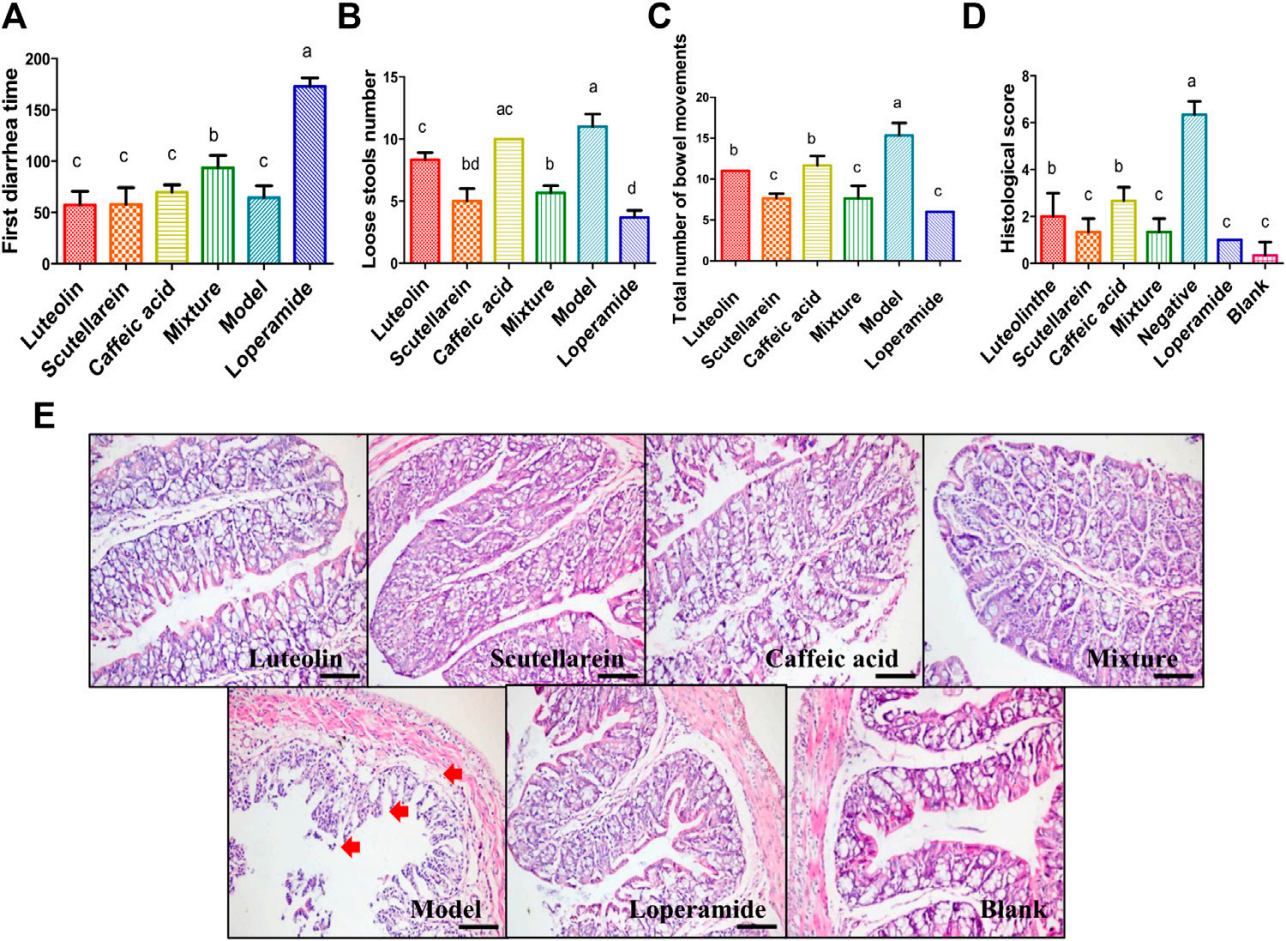
The evaluation of antidiarrhea activities of the effective components of *Plantago asiatica* L. **(A–C)** The antidiarrheal effect of three active ingredients and their mixture was compared with model and positive group. **(D)** The histological score of different groups, a high histological score indicates a serious histological injury. **(E)** The pathological section of mouse colon tissue. Results are presented as means ± SD from five groups. Different small letter superscripts in the same column indicate significant differences at *p* < 0.05.

In order to validate the antidiarrheal activity of luteolin, scutellarein, caffeic acid, and the mixture, a mouse diarrhea model was established. The results of first diarrhea time showed that the mixture of luteolin, scutellarein, and caffeic acid had significant antidiarrhea effect, while none of the three molecules had such effect alone (Figure 3A). However, both luteolin and scutellarein exhibited significant antidiarrhea effects in the results of loose stools number, total number of bowel movements, and histological score (*p*＜0.05), while caffeic acid still had no such effect in loose stools number result (Figures 3B–D). In addition, it could be observed in the pathological section results that several diarrhea-related symptoms, including intestinal epithelial cell shedding, glands and crypts disappearance, mucosal cell structure damage, submucosal hyperplasia, and vasodilation and mucosal thickness reduction, were relieved by luteolin, scutellarein, caffeic acid, and the mixture (Figure 3E). Notably, scutellarein performed similarly to the mixture, even to the positive control, in the results of loose stools number, total number of bowel movements, and histological score (Figures 3B–D).

Additionally, the loose stools inhibition rates of luteolin, scutellarein, caffeic acid, and the mixture were 24.44, 48.89, 11.11, and 55.56%, respectively. Besides, luteolin and scutellarein performed better in total number of bowel movements and histological score. Although none of the three molecules exhibited significant antidiarrhea effect in the first-time diarrhea alone, luteolin and scutellarein still had better activities than caffeic acid. Hence, luteolin and scutellarein were considered to be the main effective antidiarrheal components of *P. asiatica*. This study for the first time reported that luteolin and scutellarein had antidiarrheal activity.

### Antidiarrheal Mechanism Exploration of Luteolin and Scutellarein

**FIGURE 4:**
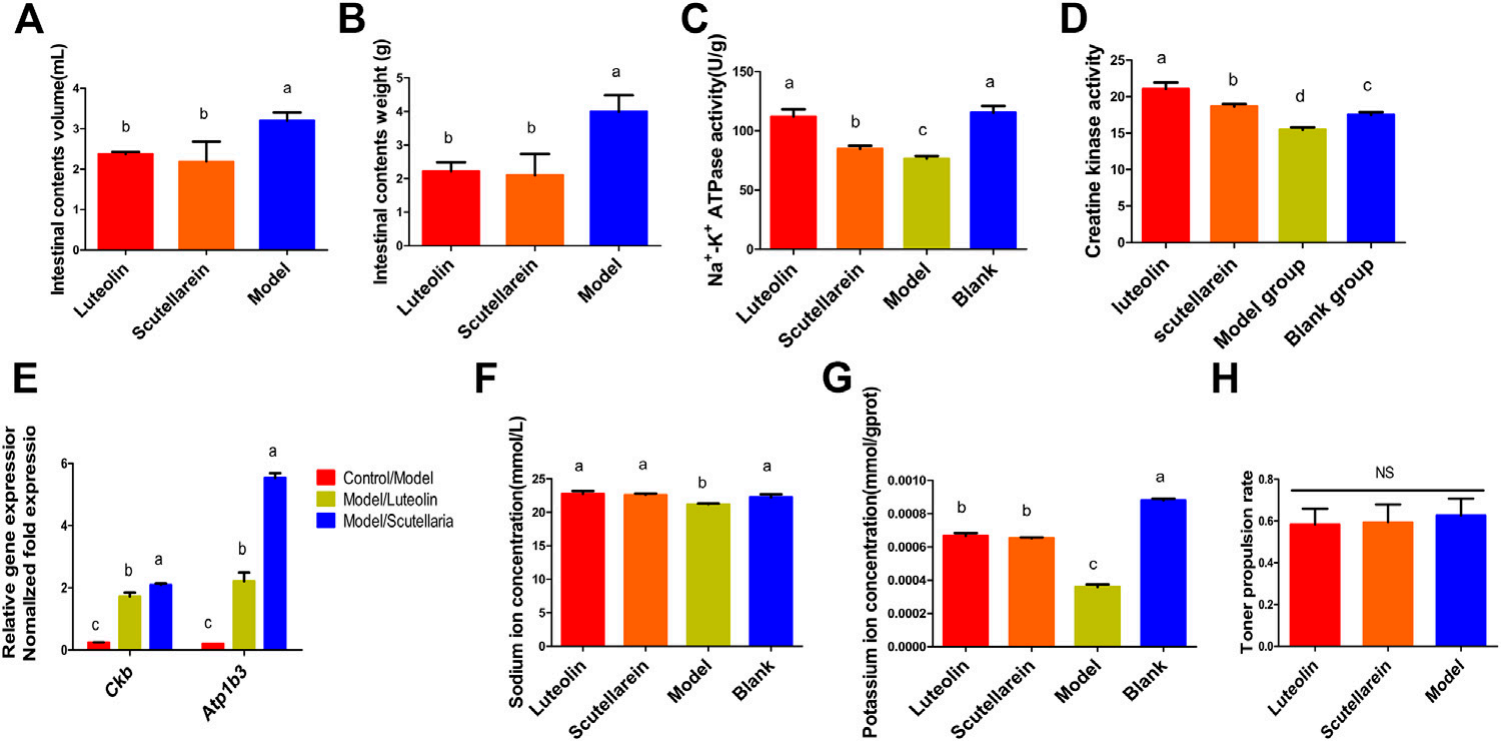
Analysis of antidiarrhea mechanism of the effective components of *Plantago asiatica* L. **(A,B)** Castor oil-induced intestinal fluid accumulation. **(C,D)** Changes in Na^+^/K^+^-ATPase and creatine kinase activities in the small intestine of rats. **(E)** The quantitative PCR. Relative gene expression>1 represents upregulating gene. In contrast, relative gene expression <1 represents a downregulating gene. **(F–G)** Changes in sodium and potassium ion concentrations in the small intestine of rats. **(H)** Charcoal meal model of intestinal motility. Data are presented as the means ± SD from five groups. Different small letter superscripts in the same column indicate significant differences at *p* < 0.05. NS indicates no significant differences.

Diarrhea caused by castor oil occurs due to the formation of ricinoleic acid in the intestinal lumen and leads to changes in intestinal homeostasis (Bakare et al., 2010), thereby increasing fluid secretion (Gurgel et al., 2002). Our data showed that the volume and weight of intestinal contents could be both significantly inhibited by luteolin and scutellarein with the inhibition rates of 25.94% and 31.88% (*p* < 0.05) (Figures 4A,B). However, compared to the model group, luteolin and scutellarein groups showed insignificant reduction in intestinal peristalsis (Figure 4H), indicating that these two ingredients did not exhibit obvious antidiarrhea effects in alleviating intestinal peristalsis, meaning the possibility of a poor combination with the opioid receptor that is consistent with other research studies (Araújo et al., 2015).

In animal intestinal cells, electrolyte balance is essential for intestinal health, and Na^+^/K^+^-ATPase can regulate the electrochemical gradients of Na^+^ and K^+^. The inhibition of Na^+^/K^+^-ATPase normal function provoked by ricinoleic acid causes the changes in the electrolyte permeability contributing to the diarrhea severity (Palombo, 2006). In addition, it reduces the activity of Na^+^/K^+^-ATPase and prevents the reabsorption of Na^+^ and K^+^ in the small intestine (Gaginella et al., 1977). As both luteolin and scutellarein could bind with Na^+^/K^+^-ATPase, the Na^+^/K^+^-ATPase activity and the Na^+^/K^+^ concentrations were determined. We found that luteolin and scutellarein could significantly upregulate both the enzyme activity and the Na^+^/K^+^ concentrations in the small intestine which were significantly downregulated in the diarrhea model group (*p* < 0.05) (Figures 4C,F,G), indicating a strong promotion function on the reabsorption of Na^+^ and K^+^ to relief diarrhea. Further studies are needed to elucidate whether luteolin and scutellarein improve Na^+^/K^+^ -ATPase activity by indirect means. CK provides temperature and space energy buffers through the CK-phosphate system to maintain cellular energy homeostasis and is responsible for providing the correct adenosine triphosphate (ATP) ATPase function, such as sodium and potassium (Na^+^/K^+^-ATPase) and hydrogen (H^+^-ATPase) pumps, resulting in changes in Na^+^ and K^+^ ion levels (Baldissera et al., 2018). Our findings showed that luteolin and scutellarein could reverse the decrease in CK activity induced by castor oil (*p* < 0.05) (Figure 4D), suggesting that luteolin and scutellarein may increase the Na^+^/K^+^-ATPase activity in a manner of upregulating CK activity.

The results of Na^+^/K^+^-ATPase and CK activities indicated that the two enzymes played important roles in the antidiarrheal activity of luteolin and scutellarein. Therefore, we further determined the gene levels of the two enzymes. According to literature reports, the expression level of *ckb* is positively correlated with the CK activity (Waskova-Arnostova et al., 2014). CASPR1 binds with *ATP1b3* and thereby contributes to the regulation of Na^+^/K^+^-ATPase maturation and trafficking to the plasma membrane in BMECs suggesting that the expression of *ATP1b3* was helpful for Na^+^/K^+^-ATPase (Zhang et al., 2019b). As the results showed, the relative gene expression of *ckb* and *Atp1b3* was significantly upregulated by luteolin and scutellarein (*p* < 0.05) (Figure 4E), illustrating that luteolin and scutellarein modify the activities of Na^+^/K^+^-ATPase and CK by regulating gene expression level.

### Preparation and Characterization of *P. asiatica* L. Superfine Powder

#### Particle Size Measurement and Appearance Quality Evaluation of *P. asiatica* L. Superfine Powder

**FIGURE 5:**
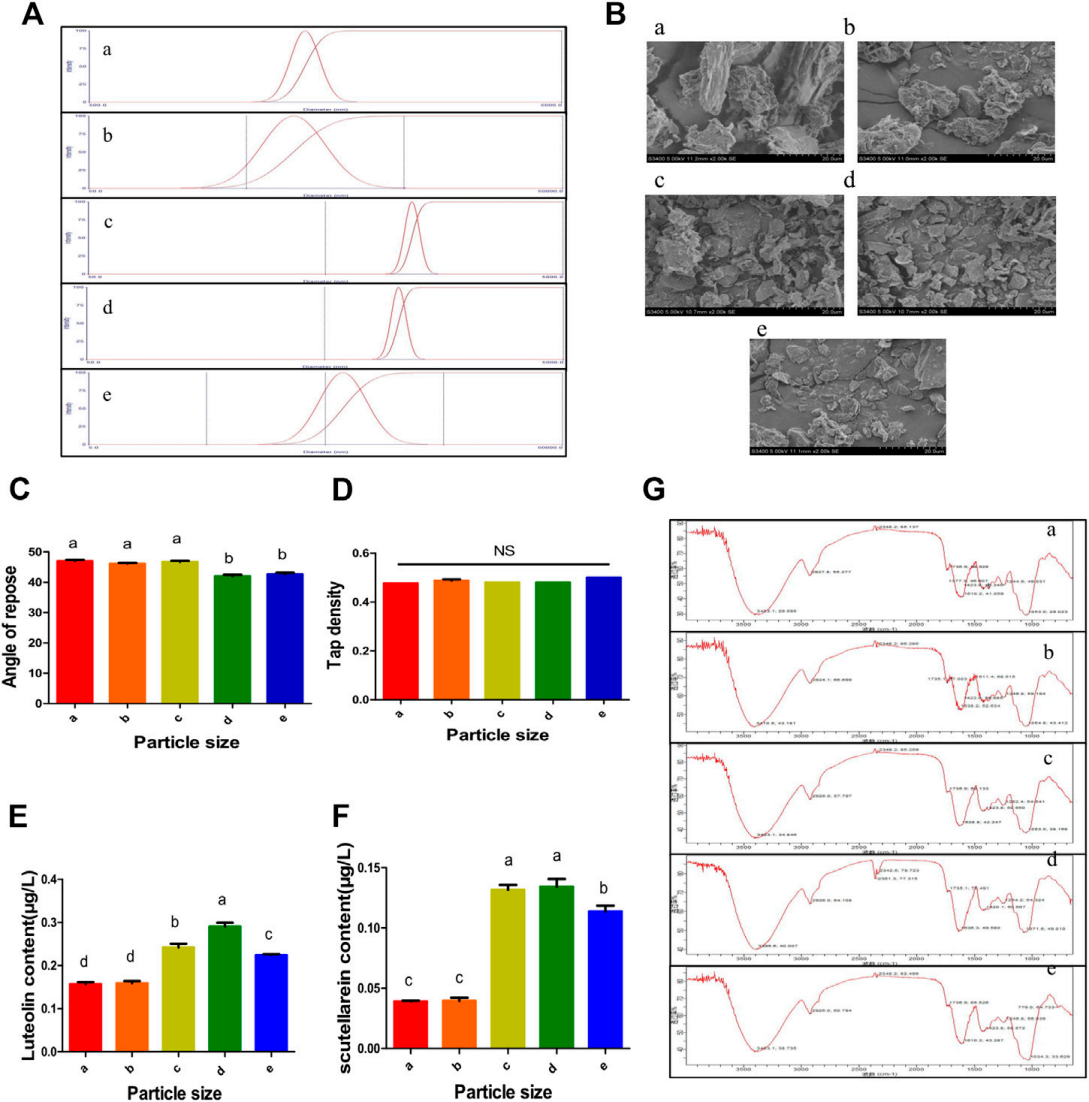
Evaluation of different characteristics of PSP. **(A)** The particle of different PSPs was determined by laser diffraction instrument. α-ε represents the particle size distributions of five different kinds of PSPs, respectively. **(B)** Different sized PSP powders were observed by 2,000× SEM. The particle size of PSP was gradually decreased from α to ε. **(C,D)** The angle of repose and tap density of different particles of PSP were determined. **(E,F)** The content of luteolin and scutellarein. **(G)** The PSP with different particles was determined through Fourier infrared spectroscopy. The numbers in the picture represent the peak absorption of each functional group in the PSP. All data are presented as means ± SD from five groups. Different small letter superscripts (a–e) in the same column indicate significant differences at *p* < 0.05.

PSP was prepared in the study, and the particle size was evaluated by laser diffraction instrument, optical microscopy, and SEM. SEM results were chosen for better accuracy. Single distribution peak in the range of 0–5,000 μm could be observed in all five experiment groups (Figure 5A); among which, the particle size distribution range of 5.27 and 3.55 μm groups was narrower than other groups, indicating a relatively uniform particle size distribution. As the particle size decreased, the particle size distribution became more concentrated. However, the last group showed an unequal result that was inconsistent with the past study (Niu et al., 2017). This might be due to the gravitational effect between tiny particles.

#### Observation on the Microscopic State of *P. asiatica* L. Superfine Powder

The PSPs with different sizes were observed under optical microscope and SEM (Supplementary Figures S1,S2 and Figure 5B), which showed that the particle sizes were gradually reduced and eventually reached a steady state. In addition, the average particle sizes were measured to be 6.66, 6.04, 5.27, 3.55, and 2.95 μm, respectively, and as the particle size decreased, the yield gradually increased and the color changed greatly while the exterior and touch hardly changed (Table 4).

**TABLE 4:** The particle determination and appearance quality evaluation of different PSPs.

Particle (μm)	α	β	γ	δ	ε
Laser particle	5.33 ± 0.516^a^	4.62 ± 0.218^b^	4.06 ± 0.082^c^	3.37 ± 0.034^d^	2.47 ± 0.020^e^
Microscope	5.64 ± 0.966^a^	4.67 ± 1.837^b^	4.06 ± 1.156^c^	3.02 ± 1.333^d^	2.18 ± 1.135^e^
SEM	6.66 ± 1.056^a^	6.04 ± 1.460^b^	5.27 ± 0.690^c^	3.55 ± 0.688^d^	2.95 ± 0.519^e^
Yield	78.03%	88.56%	88.14%	92.80%	93.63%
Exterior	Powdery	Powdery	Powdery	Powdery	Powdery
Color	Light yellow	Light yellow	Khaki	Brownish yellow	Grayish white
Touch	Fine powder feeling, no obvious graininess	Fine powder feeling, no obvious graininess	Fine powder feeling, no obvious graininess	Fine powder feeling, no obvious graininess	Very fine powder, no graininess

The different letter superscripts including α-ε indicate the particle from 6.66 to 2.95 μm and a-e indicate significant differences at *p* < 0.05.

#### The Evaluation for the Tap Density and the Angle of Repose of *P. asiatica* L. Superfine Powder

It was found in this study that the reduction of repose angle was accompanied by size decrease, indicating a better flow capacity and surface adhesion (Figure 5C). Due to the decrease in PSP particle size, the repose range decreased from 46.92° to 41.99° and the color changed from light yellow to grayish white (Table 4). According to the 2015 edition of the Chinese Veterinary Pharmacopoeia, the powder would perform general flow capacity with the repose angle ranging from 30° to 45°. As the repose angle decreased, the flow capacity of granular and the surface adhesion would be better, while the PSP would have better uniformity and inseparability (Huang et al., 2018). The differences in color might be attributed to the changes in medicinal material and water content. Moreover, a significant change in tap density was not observed (Figure 5D), and the main reason might be due to the small PSP particle size (Zhao et al., 2010).

#### Chemical Structure Analysis of *P. asiatica* L. Superfine Powder

The infrared spectra were used to measure the structures of PSPs with different sizes, which showed the infrared spectrum of the main components of each powder and the similar changing trend of different particle sizes (Figure 5G). The peak values of different functional groups are listed in Table 5. According to the results of infrared spectra, the spectra of PSPs with different particle sizes were similar, and no new chemical group bands were produced in PSPs, indicating that the main structure of PSPs was retained. However, as the particle size of PSP decreased from 6.66 to 3.55 μm, the stretching vibration of C-O, C-N, and C-O gradually increased, with peak values of 1636.3, 1254.2, and 1071.6 cm^−1^. According to other reports (Balogun et al., 2018), the absorption of *P. asiatica* at 1,601 cm^−1^ was positively correlated with the content of protein, the absorption around 1,257 cm^−1^ was related to oil and fat compounds, and the absorption at 1,051 cm^−1^ represented the stretching vibration absorption of the C–O bond in oligosaccharides, fatty acids, etc. Additionally, the absorption of these functional groups was related to the contents of certain medicinal ingredients, and stronger absorption may have better efficacy.

**TABLE 5:** Wave number assignments of FTIR spectra of PSPs with different sizes.

Group (cm^−1^)	Hydroxyl absorption	C-H key stretching vibration frequency	Carbonyl	Amide I with C-O stretching vibration	C-N stretching vibration, N-H bending vibration	C-O stretching vibration or phosphoric acid carrier absorption
α	3403.1	2927.8	1736.9	1610.2	1244.9	1053.0
β	3410.5	2924.1	1735.1	1638.2	1246.8	1054.8
γ	3403.1	2926.0	1736.9	1628.8	1252.4	1053.0
δ	3395.6	2926.0	1735.1	1636.3	1254.2	1071.6
ε	3403.1	2926.0	1736.9	1610.2	1246.8	1034.3

Afterward, the results of this study showed that the main functional groups of PSP did not change, but their peak values at 1,624, 1,264, and 1,066 cm^−1^ changed, which attributed that superfine grinding damaged the intramolecular hydrogen bonding of cellulose, but not to change the main structure of PSP (Zhao et al., 2015; Meng et al., 2018).

#### The Determination of Active Ingredient Contents of *P. asiatica* L. Superfine Powder

The contents of scutellarein and luteolin of different PSP particle sizes were determined by HPLC (Supplementary Figures S3,S4). We found that the two active ingredients increased as the size decreased from 6.66 to 3.55 μm. However, the contents decreased when the size reached 2.95 μm (Figures 5E,F), which was different from other reports (Phat et al., 2015). It is possible that there was electrostatic attraction among the particles, attracting each other when the particle size was reduced to a certain extent, which was not conducive to the dissolution of the active ingredient (Riley et al., 2018). It is generally believed that the improvement of dissolution performance primarily depends on the decrease in the particle size and the increase in the specific surface area so as to increase the contact area between the powder and the dissolution medium. However, the interaction and surface effects among the particles are prominent when the particle size is too fine, which will affect the quality of the preparation (Mullarney et al., 2011). Other than this, the medicinal materials will undergo the process of “rapid change, slow change, balance, and reverse pulverization.” The reduction of the active ingredients of 2.95 μm PSP also suggested that the comminution entered the reverse pulverization stage. All in all, the greater pulverization strength may have a greater impact on the stability and dissolution of the active ingredient of the drug. Therefore, it is necessary to determine the appropriate particle size range combining the specific properties of the drug material and dissolution in the actual application of superfine pulverization (Mullarney et al., 2011). Among the five particle sizes, the content of 3.55 μm particle size PSP was the highest with 6.8 × 10^–3^ μg/g (scutellarein) and 1.45 × 10^–2^ μg/g (luteolin) and was applied to further test.

### Optimization of Administration Dosage of *P. asiatica* L. Superfine Powder

**FIGURE 6:**
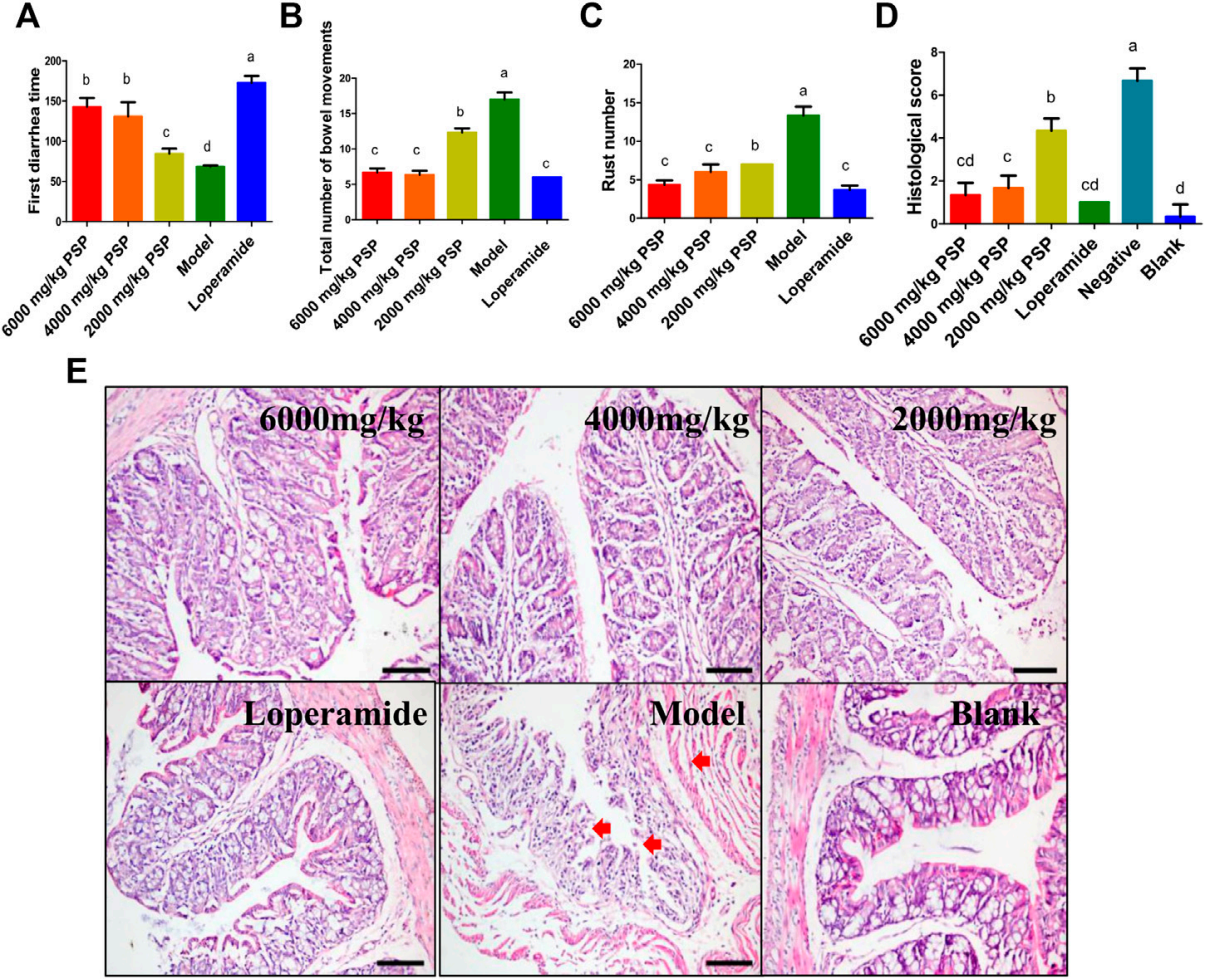
The evaluation of antidiarrhea activities of PSP. **(A–C)** Three doses of PSP groups were compared with model and positive group. Results are presented as means ± SD. Different small letter superscripts in the same column indicate significant differences at *p* < 0.05. **(D)** The histological score of different groups, a high histological score indicates a serious histological injury. **(E)** Pathological section of mouse colon tissue. Data are presented as the means ± SD from five groups. Different small letter superscripts in the same column indicate significant differences at *p* < 0.05

In order to optimize the administration dosage, the acute toxicity of PSP was first evaluated. During the observation period of 24 h, the dose of 900–10,000 mg/kg of PSP did not induce any mortality or abnormal clinical signs, and none of the tested animals showed pathological changes at the limit dose of 10,000 mg/kg, which is consistent with the research of Xiao et al. (Shang et al., 2018). Then, the antidiarrhea effects of high, medium, and low dosages were evaluated using the diarrhea model established above. Compared to the model group, all the three dosage groups exhibited significant antidiarrhea activities in terms of first diarrhea time, the total number of bowel movements, loose stools number, and histological scores (*p* < 0.05). The loose stools inhibition rates of the three groups were, respectively, 67.67, 54.89, and 46.15%. Moreover, the high and medium dosage groups performed significantly better than the low dosage group in all the four indexes (*p* < 0.05), even similarly to the positive control group in the latter three indexes (Figures 6A–D), suggesting an obvious dose-dependent trend. In accordance with these results, histological lesions were also relieved to a larger extent in the high and medium dosage groups (Figure 6E). In short, according to the results of the four major indexes mentioned above, all the three dosage groups of PSP showed significant antidiarrheal activity in an obvious dose-dependent manner, which is in accordance with other reports (Wang et al., 2015). The minimum effective dose of PSP was determined to be 2,000 mg/kg.

## Conclusion

In a word, the antidiarrheal active ingredients of PSP were identified as luteolin and scutellarein that exert antidiarrheal activity by binding with Na^+^/K^+^-ATPase. Besides, the antidiarrheal activity of luteolin and scutellarein was found in this study for the first time. In addition, superfine technology could improve the physicochemical properties and increase the effective contents of PSP. The optimal particle sizes of PSP changed from 6.66 to 3.55 μm, and the content of 3.55 μm particle size PSP was the highest with 6.8 × 10^–3^ μg/g (scutellarein) and 1.45 × 10^–2^ μg/g (luteolin), respectively. And the lowest effective dose for antidiarrhea was 2,000 mg/kg. Therefore, PSP can be considered as a new dosage form for the treatment of diarrhea.

## Data Availability Statement

The datasets presented in this study can be found in online repositories. The names of the repository/repositories and accession number(s) can be found in the article/Supplementary Material.

## Ethics Statement

The animal study was reviewed and approved by The Institutional Animal Care and Use Committee of Northeast Agricultural University.

## Author Contributions

Y-HL and W-YD conceived and designed the project. C-LD, YQ, J-XM, W-QC, X-RC, and L-YH performed majority of the experiments. YQ and C-LD wrote the manuscript. X-YC, B-OG, NE, J-JQ, and W-XG modified the manuscript. Y-HL and W-YD supervised the paper. All authors have read and agreed to submit this manuscript for publication.

## Funding

This work was supported by the National Key Research and Development Program of China (Grant No. 2018YFD0500300), the earmarked fund for China Agriculture Research System-35 (Grant No. CARS-35), the Chinese Postdoctoral Science Foundation (Grant Nos. 2019M661246), and the National Natural Science Foundation of China (Grant No. 31802228).

## Conflict of Interest

Author J-JQ was employed by the company Beijing Centre Technology Co., Ltd. The remaining authors declare that the research was conducted in the absence of any commercial or financial relationships that could be construed as a potential conflict of interest.

## Abbreviations

PSP, *Plantago asiatica* L. superfine powder; CK, creatine kinase; ATP, adenosine triphosphate; AQP4, aquaporin 4; SEM, scanning electron microscope; FTIR, Fourier transform infrared spectroscopy
