# Supplementary material for: The Active Ingredients Identification and Antidiarrheal Mechanism Analysis of Plantago asiatica L. Superfine Powder
Source: Front Pharmacol. 2021 Jan 19;11:612478. doi: 10.3389/fphar.2020.612478 (PMC7851704; doi:10.3389/fphar.2020.612478)
Supplement: Supplementary file 1 [file datasheet1.docx]

Supplementary materials

The Active Ingredients Identification and Anti-diarrheal Mechanism analysis of *Plantago asiatica* L. Superfine Powder

Chun-Liu Dong^1, 2^ ^#^, Yue Qin^1, 2^ ^#^, Jin-Xin Ma^1, 2^, Wen-Qiang Cui^1, 2^, Xing-Ru Chen^1, 2^, Li-Ya Hou^1, 2^, Xue-Ying Chen^1, 2^, Bello-Onaghise God'spower^1, 2^, Nsabimana Eliphaz^1, 2^, Jun-Jie Qin^4^, Wen-Xin Guo^5^, Wen-Ya Ding^1,3*^, Yan-Hua Li^1, 2*^

^1^ College of Veterinary Medicine, Northeast Agricultural University, Harbin, China

^2^ Heilongjiang Key Laboratory for Animal Disease Control and Pharmaceutical Development, Harbin, China

^3^ Guangxi University of Chinese Medicine, Nanning, China

^4^ Veterinary Medicine Engineering Laboratory, Beijing Centre Technology Co., Ltd. Beijing, China

^5^ Heilongjiang Provincial Agricultural Products and Veterinary Medicine Technical Appraisal Station, Harbin, China

*Correspondence to: Doctor Wen-Ya Ding, Professor Yan-Hua Li, College of Veterinary Medicine, Northeast Agricultural University, 600 Changjiang Road, Xiangfang District, Harbin, Heilongjiang 150030, P.R. China

Tel：+86 451 55191881, E‑mail: liyanhua@neau.edu.cn (Y.-H.Li).

^#^These authors contributed equally to this work.

**Supplementary Figures**

**
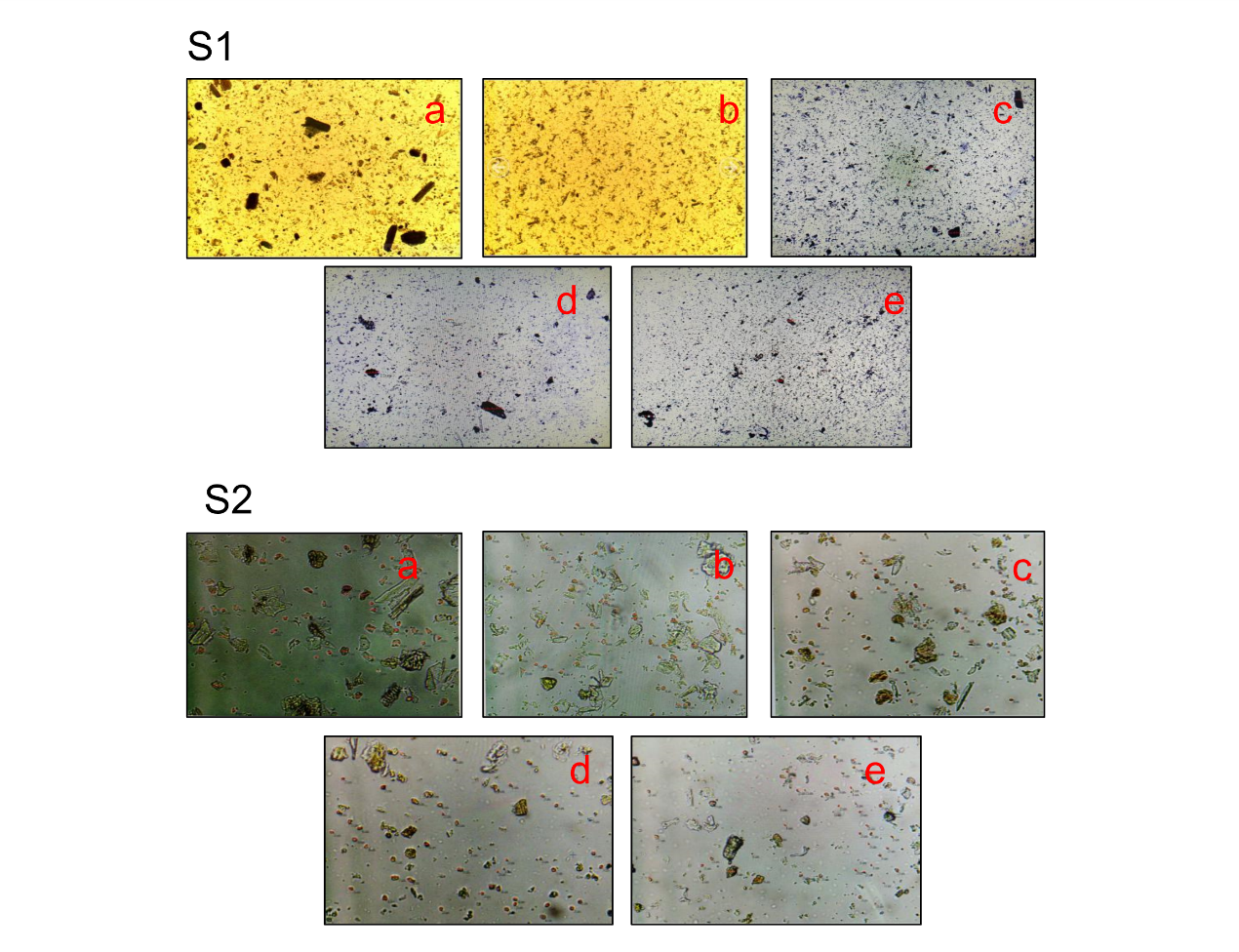
**

Figure (S1-S2) Five different particles of PSPs were observed by a 40× and 400× optical microscopy. The different particle size of PSP from a to e were gradually decreased with becoming uniform.


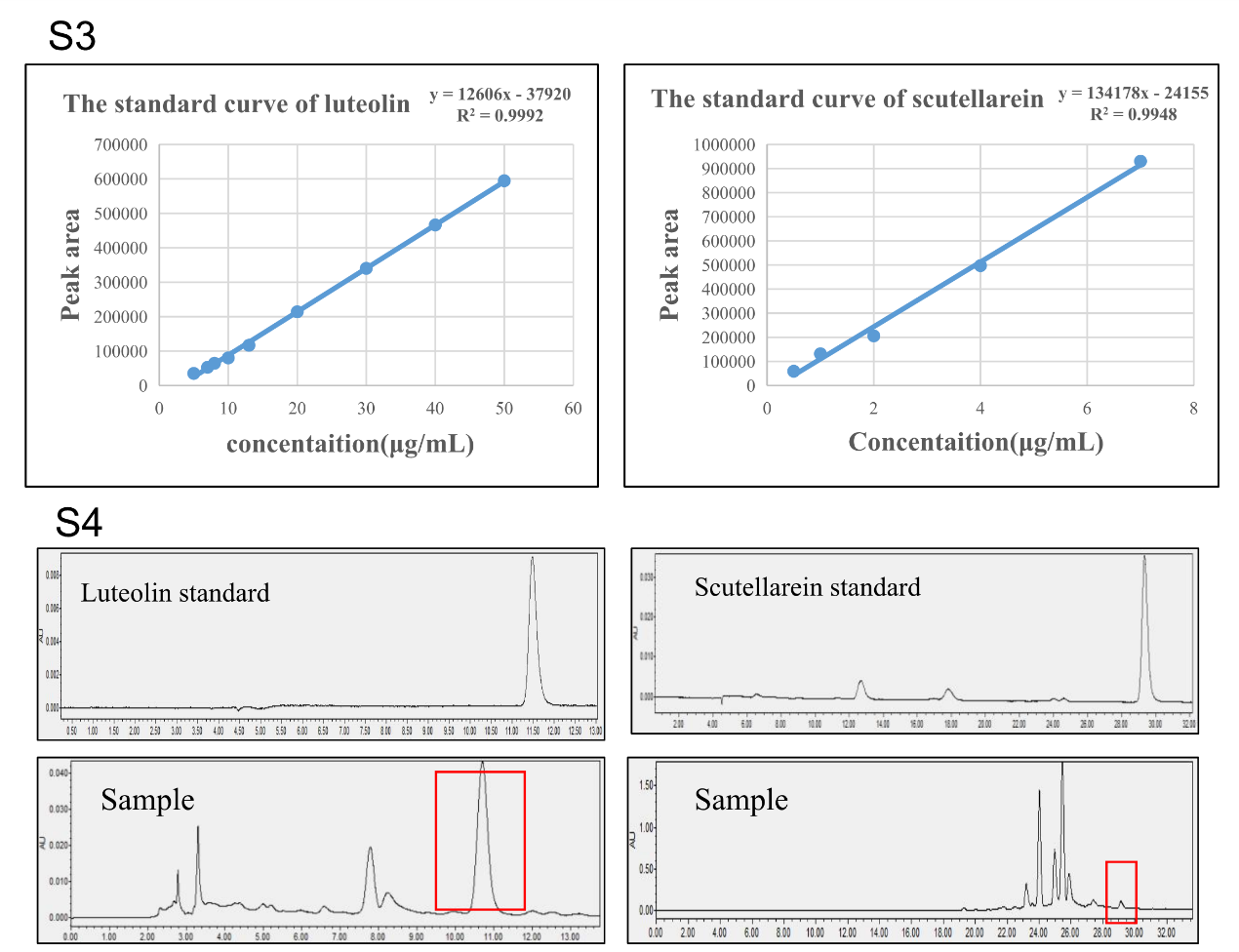


Figure (S3-S4). The content of scutellarein and luteolin in different PSPs were determined by HPLC. The standard curve of luteolin and scutellarein (S3). The Liquid chromatogram of scutellarein and luteolin and their corresponding standard (S4).
